# Transient Tumor-Fibroblast Interactions Increase Tumor Cell Malignancy by a TGF-β Mediated Mechanism in a Mouse Xenograft Model of Breast Cancer

**DOI:** 10.1371/journal.pone.0009832

**Published:** 2010-03-23

**Authors:** Christina H. Stuelten, Johanna I. Busch, Binwu Tang, Kathleen C. Flanders, Akira Oshima, Emily Sutton, Tatiana S. Karpova, Anita B. Roberts, Lalage M. Wakefield, John E. Niederhuber

**Affiliations:** 1 Cell and Cancer Biology Branch, National Cancer Institute, National Institutes of Health, Bethesda, Maryland, United States of America; 2 Laboratory of Cancer Biology and Genetics, National Cancer Institute, National Institutes of Health, Bethesda, Maryland, United States of America; 3 Laboratory of Receptor Biology and Gene Expression, National Cancer Institute, National Institutes of Health, Bethesda, Maryland, United States of America; Roswell Park Cancer Institute, United States of America

## Abstract

Carcinoma are complex societies of mutually interacting cells in which there is a progressive failure of normal homeostatic mechanisms, causing the parenchymal component to expand inappropriately and ultimately to disseminate to distant sites. When a cancer cell metastasizes, it first will be exposed to cancer associated fibroblasts in the immediate tumor microenvironment and then to normal fibroblasts as it traverses the underlying connective tissue towards the bloodstream. The interaction of tumor cells with stromal fibroblasts influences tumor biology by mechanisms that are not yet fully understood. Here, we report a role for normal stroma fibroblasts in the progression of invasive tumors to metastatic tumors. Using a coculture system of human metastatic breast cancer cells (MCF10CA1a) and normal murine dermal fibroblasts, we found that medium conditioned by cocultures of the two cell types (CoCM) increased migration and scattering of MCF10CA1a cells *in vitro*, whereas medium conditioned by homotypic cultures had little effect. Transient treatment of MCF10CA1a cells with CoCM *in vitro* accelerated tumor growth at orthotopic sites *in vivo*, and resulted in an expanded pattern of metastatic engraftment. The effects of CoCM on MCF10CA1a cells were dependent on small amounts of active TGF-β1 secreted by fibroblasts under the influence of the tumor cells, and required intact ALK5-, p38-, and JNK signaling in the tumor cells. In conclusion, these results demonstrate that transient interactions between tumor cells and normal fibroblasts can modify the acellular component of the local microenvironment such that it induces long-lasting increases in tumorigenicity and alters the metastatic pattern of the cancer cells *in vivo*. TGF-β appears to be a key player in this process, providing further rationale for the development of anti-cancer therapeutics that target the TGF-β pathway.

## Introduction

Carcinoma are complex societies of mutually interacting cells in which there is a progressive failure of normal homeostatic mechanisms, causing the parenchymal component to expand inappropriately and ultimately to disseminate to distant sites. As much as 50% or more of tumor bulk consists of non-parenchymal cells often referred to as the tumor microenvironment, including immune cells, cells of the microvasculature, and fibroblasts. Cells of the tumor parenchyma and stroma engage in extensive cross-talk, and the composition of the stroma and the nature of tumor stromal interactions change over time with tumor progression [1], [2]. Tumor-stroma cross-talk influences tumor growth by regulating angiogenesis, suppressing or subverting host immune responses, modulating extracellular matrix, and secreting signaling molecules which then in turn act on cells to further alter cell physiology and to change the composition of the cellular and acellular tumor microenvironment [2], [3], [4].

Fibroblasts are ubiquitous stromal cells which influence other cells through the secretion of cytokines and growth factors [2], [5], [6]. The response of tumor parenchymal cells to fibroblasts depends on many factors, including the nature and extent of the oncogenic lesions in the tumor cells, and the age and activation state of the fibroblasts [3], [5], [7], [8], [9].

While fibroblasts can have tumor suppressing activity the phenotype of the fibroblast changes to a tumor promoting state as carcinogenesis progresses [2]. This process occurs in two stages, generating first reversibly “primed” and then irreversibly modified fibroblasts with tumor-promoting properties [1]. Cancer-associated fibroblasts (CAF) have properties distinct from normal fibroblasts and actively promote tumorigenesis in mouse models of prostate and breast cancer [3], [10]. Typically CAFs have an activated phenotype, expressing smooth muscle actin and showing increased motility [1]. CAFs secrete tumor promoting effectors such as SDF-1, HGF, and TGF-β into the tumor microenvironment, and they overproduce extracellular matrix, which contributes to tumor rigidity and an altered signaling context thus further promoting tumor progression [5], [11], [12]. Thus, the phenotype of local fibroblasts can persistently be altered by complex reciprocal parenchymal-stromal interactions that occur during cancer progression.

TGF-βs are pleiotropic growth factors that play important roles in maintaining normal tissue homeostasis [13]. Cells release latent TGF-β into the microenvironment that upon activation binds to specific serine-threonine kinase receptors. A heterodimeric receptor complex consisting of the type II TGF-β receptor and the type I TGF-β receptor (ALK5) then activates intracellular signaling cascades that include the canonical Smad2/3 signaling path and additional pathways such as TAK1-mediated p38- or JNK signaling [14]. Dysregulation of TGF-β signaling has been implicated in the carcinogenic process in many organs. In the course of cancer progression, TGF-βs frequently switch from a tumor suppressor to a tumor promoting role [14]. In the embryo, expression of TGF-β is frequently seen at sites where critical mesenchymal-epithelial interactions occur [15], suggesting that TGF-βs may act as an important messenger between these two compartments. In support of such a role, genetic loss of response to TGF-β in fibroblasts is associated with aberrant expression of growth factors and cytokines by the fibroblasts and results in the development of premalignant and malignant lesions in several overlying epithelia in stomach, prostate and breast [8], [16], [17]. However, forced overexpression of TGF-β primes fibroblasts to promote the outgrowth of initiated human breast epithelial cells [7]. These genetic approaches imply the existence of very delicately balanced TGF-β-based homeostatic interactions between fibroblasts and the epithelial parenchyma.

When a cancer cell metastasizes, it first will be exposed to CAFs in the immediate tumor microenvironment and then to normal fibroblasts as it traverses the underlying connective tissue towards the bloodstream. Here, we have asked whether the transient interaction between tumor cells and normal fibroblasts can induce phenotypic changes in the tumor cell that then affect its malignant properties. We provide evidence that such an interaction induces the secretion and/or activation of endogenous TGF-β by the fibroblasts, which can enhance the malignant behavior of the tumor cells *in vivo*.

## Results

### Medium Conditioned by Cocultures of Fibroblasts and Tumor Cells Induces Cell Scattering in Tumor Cells

**Figure 1:**
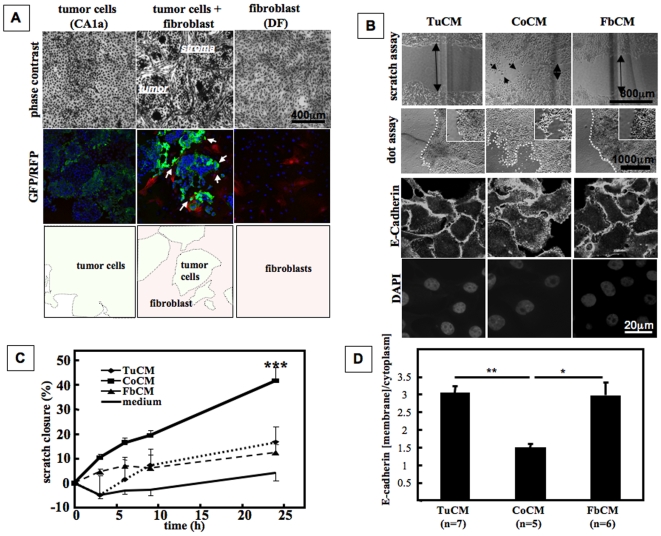
Cocultures of breast cancer cells are well organized and increase motility of tumor cells. **A.** *Upper panel*. Human breast cancer cells (CA1a) and mouse dermal fibroblasts (DF) form a well organized 2D coculture where fibroblast streaks surround tumor cell islets. *Middle panel*. Use of fibroblasts and CA1a cells that report Smad3 dependent TGF-β signaling by expression of RFP and GFP, respectively, demonstrates that TGF-β signaling in both cell types is increased in cocultures as compared to the homotypic cultures. *Bottom Panel*. Outline of the tumor cell and fibroblast compartments of the images shown in the middle panel. **B.** Medium conditioned by cocultures (CoCM) as compared to medium from homotypic fibroblast (FbCM) and tumor cell (TuCM) cultures increases cell migration and cell scattering in scratch assays (24 h) and dot assays (4 d), and induces cytoplasmic localization of E-cadherin as visualized by confocal microscopy (60 min). **C.** Influence of CoCM on closure of *in vitro* wounds (“scratch assay”). Cells were plated to confluence, mitosis inhibited by preincubation with mitomycin, and cells then stimulated with conditioned media. CoCM causes significantly faster closure of scratches as compared to all other conditioned media (scratch width after 24 h, n = 8 samples/group, ANOVA/Bonferroni; p<0.0001). **D.** Quantification of E-cadherin related immunofluorescence in CA1a cells treated with CM (Fig. 1B). Membranes and cytoplasm of cells were gated separately and average signal intensity was determined using ImageJ. Ratios of membrane and cytoplasmic signal were analyzed for statistical significance using GraphPad Prism (Kruskal-Wallis test/Dunn's Multiple Comparison Test, p = 0.0052).

We investigated the crosstalk between human metastatic breast cancer cells (MCF10CA1a hereafter referred to as CA1a) and fibroblasts (DF) in a two-dimensional coculture system. Cocultures of CA1a and DF formed an organized structure of tumor cell islets that were surrounded by elongated fibroblasts (Fig. 1A) [18]. Using CA1a cells and DF that were stably infected to report activation of TGF-β signaling by expression of fluorescent protein, we found that TGF-β signaling was activated in both tumor and stromal cells in the cocultures, as compared to much lower levels of activation in the homotypic cultures (Fig. 1A). This observation suggested that cocultures might exhibit emergent properties that were not present in the homotypic cultures. To address the effect of coculturing on secreted components of the tumor microenvironment, we used conditioned media from 4 d old homotypic cultures or cocultures to stimulate CA1a cells that had not been previously exposed to any conditioned media. In scratch assays medium conditioned by cocultures (CoCM) significantly accelerated closure of the cell-cleared area as compared to medium conditioned by tumor cells (TuCM), fibroblasts alone (FbCM), or unconditioned control medium (medium) (Fig. 1B,C). A similar result was seen when we treated tumor cells that had been plated as a tight colony and assessed migration of cells out of the colony (dot assay). This assay format more closely mimics the migration of tumor cells out of a tumor into the surrounding environment, and eliminates the wounding aspect of the scratch assay. In dot assays CoCM caused scattering of tumor cells from the edges of the colony, whereas colonies treated with homotypic media retained a defined closed edge (Fig. 1B). While CoCM increased migration of CA1a cells, proliferation of CA1a cells was unaffected by treatment with CM (Fig. S1A). At the cellular level, CoCM caused translocation of E-cadherin to the cytoplasm of CA1a cells whereas E-cadherin remained localized at the cell membrane of CA1a cells stimulated with TuCM or FbCM (Fig. 1B, D). Taken together, these data suggest that coculture of tumor cells with fibroblasts causes secretion of a soluble factor that increases cell scattering and migration of tumor cells.

### Cell Scattering Induced by Coculture Conditioned Medium Depends on Active TGF-β Synthesized by Fibroblasts

**Figure 2:**
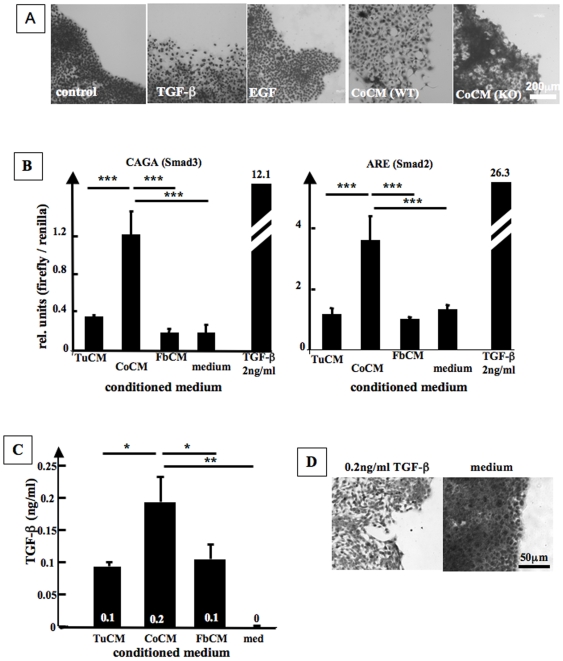
TGF-β signaling induces scattering of CA1a cells. **A.** Exogenous TGF-β and fibroblast-released TGF-β induces cell scattering in dot assays. Cells were plated into dot assays, incubated over night, and then stimulated with TGF-β (5 ng/ml), EGF (100 ng/ml), or vehicle (Control) for 4 d. Use of CoCM that was derived from cocultures of CA1a cells and TGFβ1 knockout fibroblasts (CoCM(KO)) did not stimulate cell scattering while conditioned medium from cocultures employing TGF-β1 wildtype fibroblasts (CoCM(WT)) did induce cell scattering. **B.** CoCM as compared to TuCM and FbCM induces higher levels of CAGA (Smad3) and ARE (Smad2) mediated luciferase activity in CA1a cells (n = 3, ANOVA/Dunnett's Multiple Comparison, p = 0.0002 (ARE) and p<0.0001 (CAGA)). **C.** CoCM contains higher levels of TGF-β than TuCM and FbCM. CCL-64 cells were stimulated with CM overnight and PAI-driven luciferase activity was measured. **D.** Scattering of CA1a cells is induced by addition of 0.2 ng/ml TGF-β to medium. Cells were plated into dot assays, incubated overnight, and then stimulated with TGF-β (0.2 ng/ml) or vehicle for 4 d.

Cell scattering can be induced by a variety of cytokines and growth factors [19], [20], [21], [22], [23], [24], [25], [26], and so we tested the effect of candidate factors in the dot assay. Only TGF-β (5 ng/ml, Fig. 2A) but not other growth factors such as EGF (Fig. 2A, 25 ng/ml to 250 ng/ml), TNF-α (1 ng/ml to 100 ng/ml), HGF (5 ng/ml to 20 ng/ml), latency associated protein (LAP, 1 ng/ml to 100 ng/ml) or activin A (0.1 ng/ml to 10 ng/ml), induced scattering of CA1a cells in dot assays. To address whether endogenous TGF-β might be the factor in CoCM that induces scattering, we used two different Smad-reporter systems to analyze if CA1a cells respond to TGF-β in the CM and to sensitively report on the presence of active TGF-β in the conditioned media. CoCM induced higher Smad3- and Smad2-mediated luciferase activity than did TuCM, FbCM, or medium, demonstrating that CoCM derived TGF-β increases TGF-β signaling in tumor cells (Fig. 2B). Furthermore, CoCM contained higher levels of active TGF-β (194±39 pg/ml) than the other conditioned media (TuCM: 94±6 pg/ml, FbCM: 106±22 pg/ml, medium: 0 pg/ml, Fig. 2C) as assessed by activation of a PAI-luciferase reporter activity in CCL64 cells.

We next asked if TGF-β can induce scattering of CA1a cells in concentrations similar to those secreted by cocultures into the CoCM. To do this we stimulated CA1a with medium containing 0.2 ng/ml TGF-β and observed that that this low level of TGF-β was sufficient to induce tumor cell scattering (Fig. 2D). Likewise, Smad3 driven GFP expression of the CAGA∼GFP reporter was increased by stimulation of CA1a cells with 0.2 ng/ml TGF-β (Fig. S2A). These results indicate that the concentration of TGF-β that is secreted by cocultures into the culture medium (CoCM) is sufficient to stimulate cell scattering of CA1a cells.

To ask whether this effect was limited to CA1a cells or was more universal we stimulated MCF-7 cells and T47D cells which form adherent cell colonies in culture with 0.2 ng/ml TGF-β or CoCM. Both, 0.2 ng/ml exogenous TGF-β and CoCM, were capable of inducing scattering of MCF-7 and T47D cells (Fig. S3), indicating that low concentrations of TGF-β as found in CoCM can increase motility of several breast cancer cell lines.

Since tumor cells as well as fibroblasts can secrete TGF-β, we went on to identify the source of TGF-β in our coculture system. While medium obtained from cocultures of CA1a cells with embryonic fibroblasts derived from wild type mice stimulated scattering of CA1a cells, coculture medium from CA1a cells with embryonic fibroblasts derived from TGF-β1-null mice did not induce scattering of CA1a cells (Fig. 2A). Taken together, these data suggest that coculture of tumor cells with fibroblasts induces production or activation of TGF-β1 by fibroblasts that then stimulates scattering of the tumor cells.

### CoCM Stimulates Canonical and Noncanonical TGF-β Signaling in CA1a Cells

**Figure 3 pone-0009832-g003:**
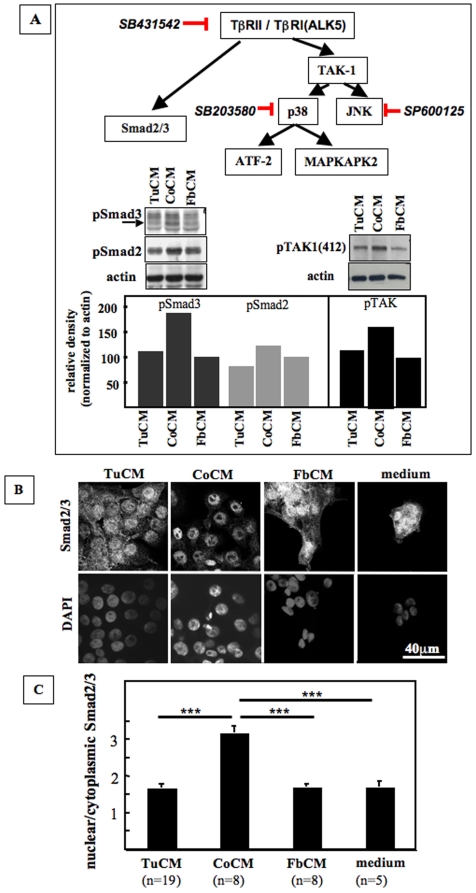
CoCM activates Smad2/3-signaling and the MAPK signaling cascades p38 and JNK in CA1a cells suggesting activation of canonical and noncanonical TGF-β signaling by CoCM derived TGF-β. **A.** TGF-β activates canonical signaling via Smad2/3 and non-canonical signaling via TAK-1 relayed JNK- and p38 signaling cascades. CoCM stimulated both, phosphorylation of TAK1, and phosphorylation of Smad2/Smad3. Cells were incubated with CoCM for 30 min (pTAK1) or 60 min (pSmad2, pSmad3). **B.** CoCM as compared to TuCM and FbCM induces nuclear localization of Smad2/3 within 60 min, indicating activation of canonical Smad signaling upon stimulation with CoCM. **C.** Quantification of Smad2/3 mediated immunofluorescence in CM treated CA1a cells (Fig. 2B). Nuclei and cytoplasm of cells were gated separately and average signal intensity was determined using ImageJ. Ratios of nuclear and cytoplasmic signal intensities were analyzed for statistical significance using GraphPad Prism (ANOVA/Dunnett's Multiple Comparison Test, p<0.0001, n = number of cells analyzed).

**Figure 4 pone-0009832-g004:**
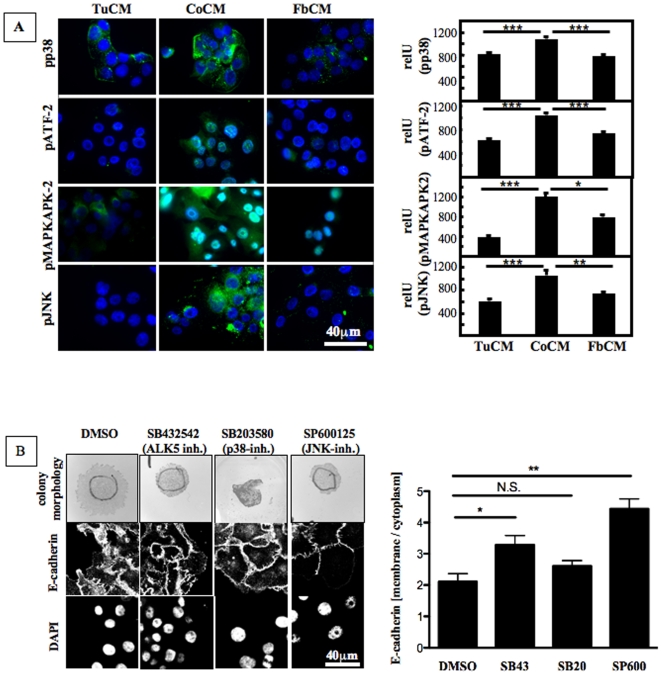
CoCM activates the MAPK signaling cascades p38 and JNK signaling in CA1a. **A.** Stimulation of CA1a cells with CoCM as compared to FbCM and TuCM for 60 min increases levels of phospho-p38 (pp38), pATF-2, pMAPKAPK-2, and pJNK as visualized by fluorescence microscopy, indicating that CoCM activates these non-canonical TGF-β signaling cascades. For quantification of pp38-, pATF-2-, pMAPKAPK2- and pJNK mediated immunofluorescence cells were gated and average signal intensity was determined using ImageJ. Ratios of nuclear and cytoplasmic signal intensities were analyzed for statistical significance using GraphPad Prism (Kruskal-Wallis test/Dunn's Multiple Comparison Test, p<0.0001 [pp38], p<0.0001 [pATF-2], p<0.0001 [pMAPKAPK2], p<0.00001 [pJNK]). 30 to 40 cells were analyzed per group. **B.** Inhibitors were added to cultures 30 min prior to stimulating CA1a cells with CoCM for 60 min. E-cadherin was visualized by immunocytochemistry and confocal microscopy. Nuclei were stained with DAPI. For quantification of E-cadherin mediated the cell membrane and the cytoplasm were gated for each cell and average signal intensities were determined using ImageJ. Ratios of nuclear and cytoplasmic signal intensities were analyzed for statistical significance using GraphPad Prism (ANOVA/Dunnetts's Multiple Comparison Test, p<0.0001. n  =  number of cells analyzed in each group.

The TGF-β signaling network includes canonical signaling via Smad2/3 as well as non-canonical signaling cascades. Non-canonical TGF-β signaling via MAPK pathways can be mediated by TAK1 that then activates JNK- and p38-signaling (Fig. 3A) [27], [28], [29]. Western blot analysis showed that Smad2, Smad3 and TAK1 were phosphorylated to a greater extent by stimulation of CA1a cells with CoCM than by stimulation with TuCM or FbCM (Fig. 3A), indicating activation of both canonical and noncanonical TGF-β signaling by CoCM. On a cellular level, CoCM induced nuclear translocation of Smad2/3in CA1a cells as demonstrated by immunostaining (Fig. 3B,C). This and the induction of Smad2 and Smad3 dependent luciferase activity (Fig. 2B) further demonstrate that CoCM activates canonical TGF-β signaling in CA1a cells. Similarly, immunofluorescence showed significantly increased levels of proteins of the p38 signaling cascade (phospho-p38, phospho-ATF-2, and phospho-MAPKAPK2) and of phospho-JNK in CA1a cells stimulated with CoCM as compared to CA1a cells stimulated with TuCM or FbCM (Fig. 4A,B). These data demonstrate that canonical and non-canonical TGF-β signaling cascades are activated upon stimulation of CA1a with CoCM.

Likewise, medium containing 0.2 ng/ml TGF-β induced increased levels of pSmad2, pTAK1, MKK3/6, pJNK, pp38, pATF-2, and pMAPKAPK2 in Western blotting (Fig. S2B), indicating that the TGF-β concentration that is found in CoCM (0.2 ng/ml) can not only stimulate cell scattering (Fig. 2D), but also is sufficient to activate canonical and non-canonical TGF-β signaling cascades in CA1a cells.

We next asked if TGF-β signaling is necessary for CoCM induced cell scattering and blocked ALK5, JNK, and p38 signaling during stimulation of CA1a with CoCM. The ALK5 inhibitor SB431542, the JNK inhibitor SP600125 and the p38 inhibitor SB203580 (Fig. 4B) all abolished tumor cell scattering induced by CoCM. On a cellular level, those same inhibitors reduced cytoplasmic translocation of E-cadherin induced by CoCM (Fig. 4B), again indicating that both canonical and noncanonical TGF-β signaling cascades are involved in CoCM induced cell scattering. Hence, CoCM stimulates tumor cell scattering via activation of both canonical and noncanonical TGF-β signaling.

### CoCM Causes Increased Tumor Growth and Metastasis *In Vivo*

**Figure 5:**
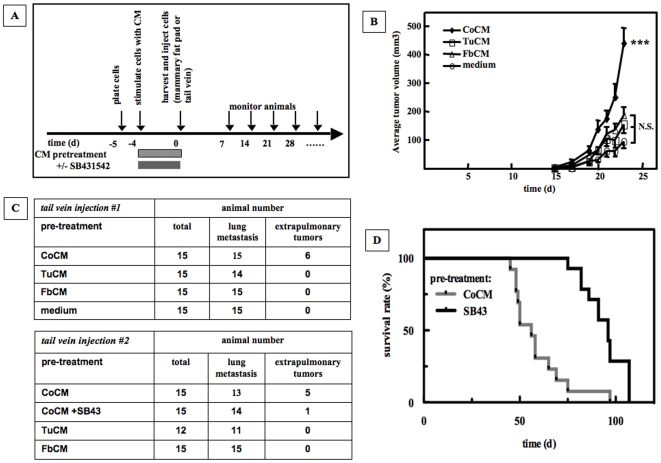
CoCM increases tumorigenicity and expands the metastatic pattern of CA1a cells. **A.** Experimental design of orthotopic implantation- and tail vein injection models. CA1a cells were pre-treated *ex vitro* with CM for 4 days, trypsinized, washed, and resuspended in DPBS. *Orthotopic Implantation Model*. 4×10^4^ cells in 50 µl DPBS were bilaterally injected into the axillary mammary fat pads of female SCID mice (15 animals or 30 tumor inoculation sites/group). Tumor size was assessed thrice weekly using calipers. Estimated tumor volumes were calculated by the formula (*S*×*S*×*L*)×0.52, where *S* and *L* are the short and long dimensions, respectively [41]. *Tail Vein Injection Model*. 2×10^5^ cells in 100 µl DPBS were injected into the tail vein of female NOD SCID mice, and animals were monitored twice weekly for signs of metastasis. **B.** *Ex vivo* pre-treatment of CA1a cells with CoCM accelerates growth of orthotopic xenograft tumors *in vivo*. The axillary mammary fat pads of female SCID mice were bilaterally inoculated with CA1a cells that were pre-treated with CM (15 animals or 30 tumor inoculation sites/group). Tumor volumes at day 23 were analyzed using GraphPad Prism 5.0. Kruskal Wallis test/Dunn's multiple comparison; p<0.001; CoCM vs TuCM: ***, CoCM vs FbCM: **, CoCM vs medium: ***, TuCM vs FbCM: N.S., TuCM vs medium: N.S., FbCM vs medium: N.S.. Representative of 2 independent experiments. **C.** *Ex vivo* pre-treatment of CA1a cells with CoCM results in extrapulmonary metastases in tail vein injection assays. CA1a cells were incubated with conditioned media for 4 days and injected into the tail vein of female NOD SCID mice. While all animals developed lung metastases, only animals injected with CoCM treated CA1a cells developed extrapulmonary tumors. Extrapulmonary tumor growth was suppressed when TGF-β signaling was inhibited by the ALK 5 inhibitor SB431542 during exposure of CA1a cells to CoCM. Animals receiving CoCM pre-treated cells had significantly higher occurrence of extrapulmonary tumors than all other groups (Chi Square Test, tail vein injection #1: p = 0.0002; tail vein injection #2: p = 0.0085). **D.** Functional TGF-β signaling of tumor cells is required for CoCM induced formation of extrapulmonary metastases. Blocking of ALK5 mediated TGF-β signaling by the ALK5-inhibitor SB435142 during *in vitro* exposure of CA1a cells to CoCM inhibited metastases and significantly increased survival time of animals *in vivo* (median survival: CoCM: 62 days (n = 13 animals), SB431542: 96 days (n = 14 animals), Kaplan Maier Analysis, p<0.0001).

Cell migration and cell scattering are *in vitro* correlates of increased tumor cell malignancy. Thus, we next asked if the transient *in vitro* exposure of CA1a cells to CoCM alters *in vivo* tumorigenicity and metastatic potential of CA1a cells. First we treated CA1a cells with CoCM for 4 d *in vitro*, and then we washed, trypsinized, and injected cells into the mammary fat pads of SCID mice (Fig. 5A). The *ex vivo* pretreatment of CA1a cells with CoCM significantly accelerated primary tumor growth at the orthotopic site when compared to CA1a cells treated with TuCM, FbCM or medium alone (Fig. 5B).

**Figure 6:**
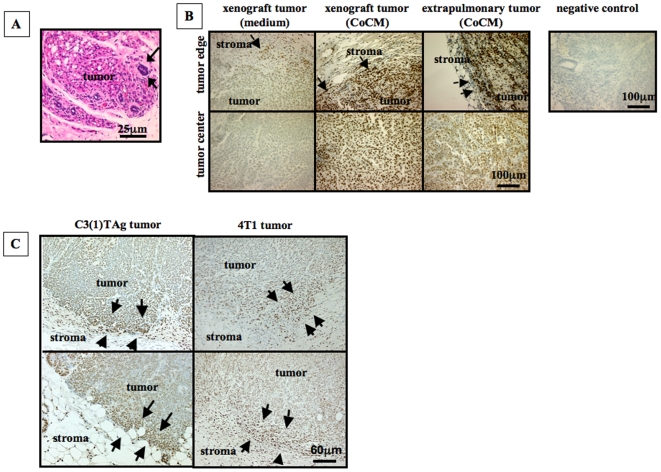
CoCM expands the metastatic pattern of CA1a cells and induces sustained TGF-β signaling via pSmad2. **A.** Extrapulmonary tumors formed by CoCM pre-treated CA1a cells after tail vein injection were highly invasive. Extrapulmonary tumors were observed in the subcutaneously, often in the proximity to mammary fat pads (arrows indicate a normal mammary duct, hematoxylin-eosin staining). **B.** Xenograft tumors and extrapulmonary metastatic tumors derived from CoCM pre-treated cells have higher levels of nuclear pSmad2 than xenograft tumors derived from medium pre-treated CA1a cells as shown by immunostaining. Levels of pSmad2 in xenograft and metastatic tumors were higher in CA1a cells invading the surrounding tissue (arrows) as compared to tumor cells in the center of tumors. The negative control was obtained by omitting the primary antibody. **C.** Nuclear pSmad2 is increased in tumor cells at the tumor stroma border (arrows) as compared to cells in the center of tumors that spontaneously developed in a C3(1)TAg model of breast cancer and in an orthotopic syngeneic model of breast cancer (4T1 cells/BALB/c mice).

The metastatic potential of CoCM treated cells was explored by injection into the tail vein of NOD SCID mice because the CA1a cell line does not metastasize efficiently from the orthotopic site. Additionally this assay format allows metastatic efficiency to be assessed independently of any effect of the experimental intervention on tumor development at the primary site. Mice in all experimental groups developed metastatic colonies of CA1a cells in the lung (Fig. 5C) and no differences of the number of lung colonies were found between the CoCM- and other treatment groups at day 40 after injections of tumor cells (Fig. S4); however, we observed a fairly high frequency of tumor colonization at extrapulmonary sites exclusively in animals that were injected with tumor cells that had been pre-treated with CoCM (Fig. 5C). Extrapulmonary tumors were seen at subcutaneous sites in the proximity of axillary and inguinal mammary fat pads, and in the thoracic wall. Histologically, subcutaneous metastatic tumors resembled the xenografted primary tumors (Fig. 6A, B).

We next asked if TGF-β was the component of the CoCM that was responsible for the expanded metastatic range of CA1a cells treated with CoCM. To do this we included the ALK5 kinase inhibitor SB431542 during the 4 day pretreatment of CA1a cells with CoCM. After washing away the SB431542 together with the CoCM, the CA1a cells were injected into NOD SCID mice using the same experimental design as before. *Ex vivo* incubation of CA1a cells with CoCM and SB431542 as compared to CoCM, resulted in significantly increased survival rates in tail vein metastasis assays (Fig. 5D). Again, we observed that animals injected with CoCM treated cells or with CoCM + SB435142 treated cells equally developed lung metastases (Fig. 5C), and when re-plated in regular culture medium, proliferation of CA1a cells pre-treated with CoCM was equal to proliferation of CA1a cells pretreated with CoCM +SB431542, indicating that SB431542 did not negatively influence proliferation or survival of pre-treated cells (data not shown). However, only 1/14 animal injected with CoCM + SB431542 developed an extrapulmonary tumor, while 5/15 animals that were injected with CoCM treated tumors developed extrapulmonary tumors in tail vein assays (Fig. 5C). Thus, a transient *in vitro* exposure of CA1a cells to CoCM and consequent activation of TGF-β signaling in these cells not only durably increases tumorigenicity but also permits successful colonization of a wider range of target tissues.

### Active TGF-β Signaling Is Sustained in Tumors Originating from CoCM Treated Cells

We next asked if TGF-β signaling in CA1a cells is durably increased *in vivo* after stimulation of CA1a cells with TGF-β containing CoCM *in vitro*. Nuclear pSmad2 levels were higher in xenograft tumors or metastatic tumors originating from CoCM treated cells than in xenograft tumors originating from medium treated cells (Fig. 6B). Thus, increased TGF-β signaling in CA1a cells by treatment with CoCM is sustained *in vivo*, indicating that the transient exposure of CA1a cells to elevated TGF-β levels causes a durable change in TGF-β signaling activity.

### TGF-β Signaling Is Increased at the Tumor - Stroma Border of Tumors in Several Mouse Models

Next, we investigated whether TGF-β signaling is increased when tumor cells invade the fibroblast-containing stroma surrounding a primary tumor as would be predicted from our observations. Immunostaining of pSmad2 in sections of mammary tumors derived from different mouse breast cancer models (CA1a xenograft tumors, syngeneic mouse mammary tumor (4T1), and tumors from the C3(1)TAg transgenic model) revealed that pSmad2 levels are higher in cells at the tumor stroma border, and particularly in those cells invading the surrounding stroma, as compared to tumor cells in the center of the tumor (Fig. 6C). Thus, activation of pSmad2 naturally occurs when tumor cells invade the surrounding stroma.

**Figure 7:**
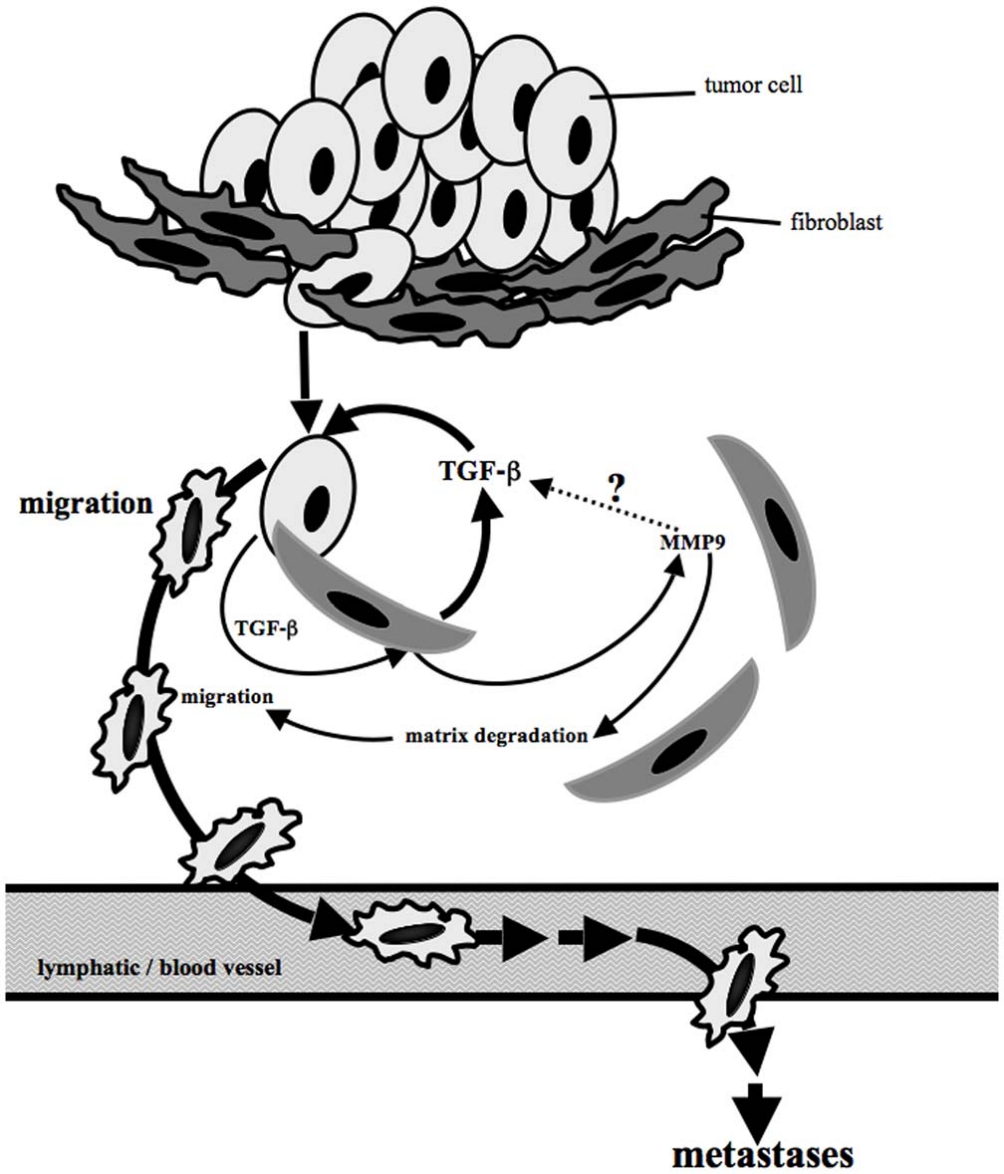
Tumor-stroma interactions increase malignancy of tumor cells by bidirectional effects of TGF-β. Direct interactions of tumor cells and fibroblasts as they occur when parenchymal tumor cells invade the underlying stroma increase secretion of TGF-β by tumor cells as well as by fibroblasts. TGF-β has bidirectional effects on both cells types. Fibroblast derived TGF-β can directly stimulate tumor cell migration and malignancy. Tumor cell derived TGF-β induces fibroblasts to secrete MMP-9 [18]. Increased MMP-9 levels then might further increase levels of active TGF-β, and also facilitate matrix degradation and migration of tumor cells, further increasing the malignant potential of tumor cells.

Taken together, our results imply that tumor cells, once they traverse through the surrounding stroma, can subvert normal fibroblasts to increase levels of active TGF-β in the microenvironment that then induces tumor cell migration, cell scattering, and an expanded metastatic pattern, thereby boosting the malignancy of tumor cells (Fig. 7).

## Discussion

Interactions between tumor cells and the fibroblasts of the tumor microenvironment are complex, reciprocal and stage-dependent [30], [31], [32]. Here we have shown that transient interactions of breast carcinoma cells with normal fibroblasts *in vitro* can increase tumor cell malignancy and expand the metastatic range of tumor cells *in vivo* by a TGF-β dependent mechanism. Specifically, the interaction of human breast carcinoma cells with normal murine fibroblasts induces aberrant secretion of active TGF-β by the fibroblasts. This transient exposure of tumor cells to fibroblast-derived TGF-β then durably increases tumorigenesis even when the two cell types are no longer interacting. Thus, tumor cells traversing through connective tissue may exploit their transient interaction with normal fibroblasts so as to get a boost in the metastatic process (Fig. 7). This phenomenon has a number of interesting features that are discussed below.

### Emergent Properties and Reciprocity in the Co-Cultures

The interaction between tumor cells and normal fibroblasts can generate emergent system properties that are not seen when the cells are cultured separately. Our experimental design allowed us to focus specifically on the impact of this interaction on the tumor cells, and to start to identify soluble mediators. We showed that co-culture of the two cell types significantly increased the levels of fibroblast derived active TGF-β in the extracellular environment, and that this TGF-β subsequently enhanced migration of the tumor cells *in vitro* and malignant behavior *in vivo*. It has been previously proposed that tumor cells can induce a reversibly “primed state” in normal fibroblasts, in which the fibroblasts can promote tumorigenesis even though they do not have all the characteristics of the irreversibly modified CAFs [1]. Our data suggest that such priming may involve cancer cell-induced production of active TGF-β by the fibroblasts. It is likely that TGF-β produced by these primed fibroblasts contributes to the elevated TGF-β levels that are seen in the vicinity of many advanced human tumors.

Co-cultures of fibroblasts and tumor cells showed increased TGF-β signaling in both cell compartments, consistent with the presence of activated TGF-β in the CoCM (Fig. 1A). We have previously shown that TGF-β derived from the tumor cell compartment in this co-culture model can induce MMP-9 secretion by fibroblasts in a Smad-, ras- and PI3K dependent fashion [18]. In the present work, we demonstrated that TGF-β derived from the fibroblast compartment enhanced the migration of tumor cells through the activation of Smad-, p38- and JNK-signaling cascades. Together the data suggest that TGF-β-mediated signaling between the tumor cells and fibroblasts is bidirectional, involves a distinct combination of signaling cascades in each partner, and has different molecular outcomes in each compartment. It is currently not clear how the two cell types can selectively respond to the TGF-β produced by the other, but this phenomenon conceivably might reflect differences in TGF-β isoforms, and/or structure of the latent complexes made by each cell type, as well as cell specific regulation of signal flux through the TGF-β network within each cell type. However, since MMP-9 can create a microenvironment more favorable to invasion and metastasis through activation of growth factors, specifically TGF-β, and destruction of extracellular matrix [33], the combined biological outcome of the differing effects of TGF-β on each compartment is likely to be a further enhancement of malignant behavior (Fig. 7).

### Transient Tumor-Fibroblast Interactions Generate Small Magnitude Molecular Effects That Induce Major Changes in Outcome

Exposure of rat mammary tumor cells to exogenous TGF-β *ex vivo* has previously been shown to promote metastatic efficiency *in vivo* [34]. Here we have shown that transient interactions between tumor cells and normal fibroblasts, such as might occur when tumor cells traverse the surrounding tissue, can have a similar effect. The amount of active TGF-β that was produced by the coculture was very low, and the activation of downstream signaling pathways was similarly mild (Figs 2 to [Fig pone-0009832-g003] [Fig pone-0009832-g004] 5). Nevertheless, these small magnitude, endogenous molecular effects altered the phenotype of the tumor cell such that it showed significantly increased malignant behavior even when no longer in contact with the fibroblast compartment. The data imply that carcinoma cells that have breached the basement membrane and are migrating through underlying connective tissue may be able to subvert and use resident, TGF-β secreting fibroblasts to boost their motility and subsequent metastatic spread.

### Underlying Mechanisms

TGF-β has a number of tumor cell-targeted effects that could contribute to the enhanced malignancy, including enhancement of tumor cell migration and invasion, direct effects on tumor cell survival, and changes in the composition of secreted growth factors and extracellular matrix components [14], [35]. The stimulatory effect of CoCM on primary tumor growth may reflect pro-survival effects of TGF-β on CA1a cells as TGF-β reduces apoptosis of CA1a cells (Fig. S1B), possibly acting in concert with other as yet unidentified trophic factors in the CM.

The ability of TGF-β to promote migration and invasion of CA1a cells may contribute to the enhanced metastasis, but additional mechanisms such as altered expression levels of junctional proteins like E-cadherin, integrins, or chemokines and chemokine receptors are also likely to play a role [36]. For example, the organ tropism of metastatic tumors is determined in part by the spectrum of chemokines and chemokine receptors that they express [37], and TGF-βs have been shown to modulate relevant chemokine ligand/receptor axes in other models. TGF-β derived from cancer-associated fibroblasts upregulates CXCR4 in initiated prostatic epithelial cells [38], and expression of CXCR4 correlates with lymph node metastasis in multiple human tumors [39]. It will be the aim of future work to determine which mechanism is relevant for the expanded metastatic range shown by the CA1a breast cancer cells stimulated with fibroblast derived TGF-β.

### Consequences and Conclusions

In conclusion, our results demonstrate that a relatively short-lived interaction between tumor cells and normal fibroblasts induces changes in the localized extracellular microenvironment that then substantially increase the malignancy of the tumor cells even after the original interaction has ceased. The data imply that normal resident fibroblasts that are exposed to direct contact with invading tumor cells may boost the motility and subsequent metastatic spread of these carcinoma cells. TGF-β appears to be a key player in this process, providing further rationale for the development of anti-cancer therapeutics that target the TGF-β pathway [40].

## Materials and Methods

### Cell Culture and Generation of Conditioned Media

MCF10CA1a (“CA1a”) is a malignant, metastatic human breast cancer line [41]. Mouse dermal fibroblasts (DF) were derived from newborn mice as previously described [42]. Mouse embryonic fibroblasts were derived from embryos (ED 12 to ED 14) by suspending embryonic tissue in DMEM (Invitrogen, Carlsbad, CA) supplemented with 10% fetal calf serum (“fibroblast medium”). CA1a and DF were respectively grown in DMEM/F12 supplemented with 5% horse serum (“CA1a medium”) and DMEM, low glucose (Invitrogen, Carlsbad, CA), supplemented with 10% fetal bovine serum (Invitrogen or Gemini Bio-Products, Woodland, CA) [18]. To obtain conditioned media from homotypic cultures or cocultures, 1.5×10^6^ cells of each type were plated in a 150 cm^2^ dish and incubated in a 1∶1 mixture of CA1a- and fibroblast medium (“coculture medium”). Culture supernatants were collected after 4 d and cells were removed by centrifugation (200 g, 5 min). Acellular supernatants were diluted with fresh coculture medium (conditioned medium, CM) and used for further experiments [18].

### Cell Scattering and Migration

#### Dot Assay

To assess cell scattering, 20 µl of cell suspension (3×10^6^ CA1a cells/ml) were placed in each well of 6-well plates and allowed to adhere to form tight well-circumscribed colonies. After 3 hours non-attached cells were removed by rinsing the plate with calcium-containing Dulbecco's phosphate buffered saline (DPBS, Invitrogen, Carlsbad, CA). Cells were incubated in regular culture medium overnight, and then switched for 4 days to a 1∶1 mixture of CM and fresh coculture medium. Cell scattering was evaluated macroscopically and microscopically following staining with hematoxylin-eosin. Unconditioned medium served as an additional negative control. The effect of growth factors and cytokines on cell scattering was assessed by adding the following growth factors in concentrations as indicated to the culture medium for 4 days: human TGF-β1, human EGF, LAP (all R&D Systems, Minneapolis, MN), human TNF-α (PeproTech, Rocky Hill, NJ), and HGF (kind gift from Don Bottaro). Where indicated, small molecule inhibitors of various signaling pathways (ALK4,5,7: SB431542, 5 µM, Sigma, St. Louis, MO; MEK/Erk: PD98059, 25 µM; p38: SB203580, 10 µM; JNK: SP600125, 10 µM (all Calbiochem, San Diego, CA)) were added to cultures 30 min before stimulation with the conditioned medium. Equal amounts of DMSO served as a negative control.

#### Scratch Assay

CA1a cells were plated in 24-well plates and grown to confluence. Cell proliferation was blocked by a 30 min pretreatment with mitomycin C (25 µg/ml). A scratch was then made in each well using a 100 µl pipette tip and cells were stimulated with the various CM in 1∶1 mixtures with fresh coculture medium. The scratch width at different time points was imaged and measured using ImagePro. Data were analyzed using GraphPad Prism 2.0.

### Immunostaining and Western Blotting

Immunofluorescence and Western Blotting were performed following established protocols [18], [43]. The following antibodies were used: Immunofluorescence: phospho-JNK, phospho-p38, phospho-ATF2, phospho-MAPKAPK2 (all 1∶100, Cell Signaling, Danvers, MA) and E-cadherin (1∶800, Zymed, Carlsbad, CA). Western Blotting: Smad3 (1∶1000, Zymed), Smad2/3 (1∶1000, BD Biosciences, San Jose, CA), phospho-Smad3 (1∶12 000, Gift from E. Leoff), phospho-Smad2, phospho-TAK, TAK (both 1∶1000, Cell Signaling), and actin (1∶60 000, BD Biosciences) for Western Blotting. Immunoperoxidase staining [42] for phospho-Smad2 (1∶500, Millipore, Temecula,CA) was performed on formalin-fixed, paraffin-embedded tumor sections.

### Transfection, Reporter Assays, and Lentiviral Infection

Transfection of CA1a cells with the CAGA_12_-luciferase plasmid or with the reporter plasmids ARE-luciferase/FAST-1, and lentiviral infections were performed as described previously [44], [45].

### Quantification of TGF-β

Levels of active TGF-β in CM were determined using PAI-driven luciferase expression in CCL-64 cells [46]. Briefly, CCL-64 cells that were stably transfected with PAI-luc were cultured in 48-well plates (20000 cells/well) in 200 µl RPMI supplemented with 5% FCS for 36 hours and serum starved (RPMI supplemented with 1% FCS) for 8 hours. Then 100 µl medium was removed and replaced with 100 ul CM (sample) or 100 µl unconditioned medium spiked with varying concentrations of TGF-β1 to establish a standard curve. Cells were incubated over night, and luciferase assays were performed according to the manufacturer's instructions (Promega, Madison, WI).

### *In vivo* Tumorigenesis

Female SCID and NOD SCID animals (age: 8–10 weeks) were purchased from the NCI Animal Production Program. For tumorigenesis studies, 4×10^4^ tumor cells in 50 µl DPBS were bilaterally injected into the # II and # VII mammary fat pads of female SCID mice. Tumor size was assessed using calipers. Tumor volumes were calculated by the formula for an oblique spheroid (*S*×*S*×*L*)×0.52, where *S* and *L* are the short and long dimensions, respectively. Tail vein injection assays were performed by injecting 2×10^5^ cells in 100 µl DPBS into the tail vein of female NOD SCID animals [41]. Mice were euthanized after 8 weeks or when animals became moribund and examined grossly on necropsy for the presence of metastases in multiple organs. Metastasis was confirmed histologically.

Orthotopic syngeneic mammary tumors of mouse breast cancer cells (4T1) in BALB/c mice and C3(1)TAg tumors were grown as previously described [47], [48].

### Statistical Analysis

Cumulative tumor volumes [47] and survival graphs were analyzed by ANOVA (post hoc test Dunnett's Multiple Comparison Analysis), Kruskal-Wallis test (post hoc test Dunn's Multiple Comparison Analysis), Chi Square test, and Kaplan-Maier assays using Graph Pad Prism 5.0b. Data are presented as mean ± standard error of mean (SEM).

## Supporting Information

Figure S1 Influence of conditioned media and TGF-beta on cell proliferation and apoptosis. CA1a cells (2000/well) were plated into 96 well plates and allowed to attach. Cell were then stimulated with conditioned media or with TGF-beta (5 ng/ml) and incubated at 37C, 5% CO2 for another 48 h. Thymidine incorporation was performed as described earlier. BrdU incorporation and apoptosis were measured using the Cell Proliferation (BrdU) kit and the Cell Death Detection kit (both Roche) according to the manufacturer's instructions.
(0.17 MB TIF)

Figure S2 TGF-β (0.2 ng/ml) induces canonical Smad2/3 and non-canonical MAPK-signaling in CA1a cells. A. CA1a cells were stably infected with CAGA12∼GFP. Cells were serum starved overnight and then stimulated with TGF-β in the concentrations indicated. Images of confluent cultures were takes 24 h later and green fluorescence analyzed using Image J. Data points present mean + standard error of mean (SEM) of 9 field. B, C. Western blot analysis demonstrates that TGF-β (0.2 ng/ml) activates canonical and non-canonical TGF-β signaling as evident by increased levels of pSmad2, pTAK412, pMKK3/6, pJNK, pp38, pATF-2, and pMAPKAPK2; actin was used as loading control. C. Quantification of data shown in B. Densitometry of bands was performed using ImageJ, and data were analyzed in Excel.
(0.26 MB TIF)

Figure S3 Scattering of the human breast cancer cells (MCF-7 and T47D) is induced by TGF-β (0.2 ng/ml) and CoCM. MCF-7 and T47D were cultured in DMEM supplemented with 10% FCS. For experiments, cells were plated into 6-well plates, incubated overnight, and then stimulated with TGF-β (0.2 ng/ml) or CM for 4 days. Colonies were visualized by H&E staining. well plates, incubated overnight, and then stimulated with TGF-β (0.2 ng/ml) or CM for 4 days. Colonies were visualized by H&E staining
(0.51 MB TIF)

Figure S4 Pre-treatment of CA1a with CoCM does not significantly alter lung metastases. CA1a cells were pre-treated ex vitro with CM for 4 days, trypsinized, washed, and resuspended in DPBS. 2×105 cells in 100 ml DPBS were injected into the tail vein of female NOD SCID mice, and animals were monitored twice weekly for signs of metastasis. Animals were euthanized 40 d after injection of tumor cells to assess lung metastases. Lungs were fixed in 4% normal buffered paraformaldheyde. Tumor colonies were counted in cross sections of the lung (1 section/lung). Data were analyzed using GraphPad Prism (ANOVA/Dunnett Multiple comparison test). Single data points and median with interquartile range.
(0.17 MB TIF)

## References

[1] Beacham DA, Cukierman E (2005). Stromagenesis: the changing face of fibroblastic microenvironments during tumor progression.. SeminCancer Biol.

[2] Proia DA, Kuperwasser C (2005). Stroma: tumor agonist or antagonist.. Cell Cycle.

[3] Olumi AF, Grossfeld GD, Hayward SW, Carroll PR, Tlsty TD (1999). Carcinoma-associated fibroblasts direct tumor progression of initiated human prostatic epithelium.. Cancer Res.

[4] Liotta LA, Kohn EC (2001). The microenvironment of the tumour-host interface.. Nature.

[5] Baglole CJ, Ray DM, Bernstein SH, Feldon SE, Smith TJ (2006). More than structural cells, fibroblasts create and orchestrate the tumor microenvironment.. ImmunolInvest.

[6] Micke P, Ostman A (2005). Exploring the tumour environment: cancer-associated fibroblasts as targets in cancer therapy.. ExpertOpinTherTargets.

[7] Kuperwasser C, Chavarria T, Wu M, Magrane G, Gray JW (2004). Reconstruction of functionally normal and malignant human breast tissues in mice.. ProcNatlAcadSciUSA.

[8] Bhowmick NA, Moses HL (2005). Tumor-stroma interactions.. CurrOpinGenetDev.

[9] Campisi J (2005). Senescent cells, tumor suppression, and organismal aging: good citizens, bad neighbors.. Cell.

[10] Orimo A, Gupta PB, Sgroi DC, renzana-Seisdedos F, Delaunay T (2005). Stromal fibroblasts present in invasive human breast carcinomas promote tumor growth and angiogenesis through elevated SDF-1/CXCL12 secretion.. Cell.

[11] Egeblad M, Littlepage LE, Werb Z (2005). The fibroblastic coconspirator in cancer progression.. Cold Spring HarbSympQuantBiol.

[12] Paszek MJ, Zahir N, Johnson KR, Lakins JN, Rozenberg GI (2005). Tensional homeostasis and the malignant phenotype.. Cancer Cell.

[13] Massague J (2000). How cells read TGF-beta signals.. NatRevMolCell Biol.

[14] Massague J (2008). TGFbeta in Cancer.. Cell.

[15] Timme TL, Truong LD, Merz VW, Krebs T, Kadmon D (1994). Mesenchymal-epithelial interactions and transforming growth factor-beta expression during mouse prostate morphogenesis.. Endocrinology.

[16] Bhowmick NA, Chytil A, Plieth D, Gorska AE, Dumont N (2004). TGF-beta signaling in fibroblasts modulates the oncogenic potential of adjacent epithelia.. Science.

[17] Cheng N, Bhowmick NA, Chytil A, Gorksa AE, Brown KA (2005). Loss of TGF-beta type II receptor in fibroblasts promotes mammary carcinoma growth and invasion through upregulation of TGF-alpha-, MSP- and HGF-mediated signaling networks.. Oncogene.

[18] Stuelten CH, Byfield SD, Arany PR, Karpova TS, Stetler-Stevenson WG (2005). Breast cancer cells induce stromal fibroblasts to express MMP-9 via secretion of TNF-{alpha} and TGF-{beta}.. JCell Sci.

[19] Goswami S, Sahai E, Wyckoff JB, Cammer M, Cox D (2005). Macrophages promote the invasion of breast carcinoma cells via a colony-stimulating factor-1/epidermal growth factor paracrine loop.. Cancer Res.

[20] Hagemann T, Wilson J, Kulbe H, Li NF, Leinster DA (2005). Macrophages induce invasiveness of epithelial cancer cells via NF-kappa B and JNK.. JImmunol.

[21] Jechlinger M, Grunert S, Beug H (2002). Mechanisms in epithelial plasticity and metastasis: insights from 3D cultures and expression profiling.. JMammaryGlandBiolNeoplasia.

[22] Kang H, Watkins G, Parr C, Douglas-Jones A, Mansel RE (2005). Stromal cell derived factor-1: its influence on invasiveness and migration of breast cancer cells in vitro, and its association with prognosis and survival in human breast cancer.. Breast Cancer Res.

[23] Kawai N, Tsuji S, Tsujii M, Ito T, Yasumaru M (2002). Tumor necrosis factor alpha stimulates invasion of Src-activated intestinal cells.. Gastroenterology.

[24] Lewis MP, Lygoe KA, Nystrom ML, Anderson WP, Speight PM (2004). Tumour-derived TGF-beta1 modulates myofibroblast differentiation and promotes HGF/SF-dependent invasion of squamous carcinoma cells.. BrJCancer.

[25] Rosen EM, Zitnik RJ, Elias JA, Bhargava MM, Wines J (1993). The interaction of HGF-SF with other cytokines in tumor invasion and angiogenesis.. EXS.

[26] Thomas GJ, Hart IR, Speight PM, Marshall JF (2002). Binding of TGF-beta1 latency-associated peptide (LAP) to alpha(v)beta6 integrin modulates behaviour of squamous carcinoma cells.. BrJCancer.

[27] Javelaud D, Delmas V, Moller M, Sextius P, Andre J (2005). Stable overexpression of Smad7 in human melanoma cells inhibits their tumorigenicity in vitro and in vivo.. Oncogene.

[28] Wakefield LM, Roberts AB (2002). TGF-beta signaling: positive and negative effects on tumorigenesis.. Curr Opin Genet Dev.

[29] Yamaguchi K, Shirakabe K, Shibuya H, Irie K, Oishi I (1995). Identification of a member of the MAPKKK family as a potential mediator of TGF-beta signal transduction.. Science.

[30] Kopfstein L, Christofori G (2006). Metastasis: cell-autonomous mechanisms versus contributions by the tumor microenvironment.. Cell MolLife Sci.

[31] Nelson CM, Bissell MJ (2006). Of extracellular matrix, scaffolds, and signaling: tissue architecture regulates development, homeostasis, and cancer.. AnnuRevCell DevBiol.

[32] Stover DG, Bierie B, Moses HL (2007). A delicate balance: TGF-beta and the tumor microenvironment.. JCell Biochem.

[33] Liotta LA, Stetler-Stevenson WG (1990). Metalloproteinases and cancer invasion.. SeminCancer Biol.

[34] Welch DR, Fabra A, Nakajima M (1990). Transforming growth factor beta stimulates mammary adenocarcinoma cell invasion and metastatic potential.. ProcNatlAcadSciUSA.

[35] Rahimi RA, Leof EB (2007). TGF-beta signaling: a tale of two responses.. JCell Biochem.

[36] Giehl K, Menke A (2008). Microenvironmental regulation of E-cadherin-mediated adherens junctions.. Front Biosci.

[37] Muller A, Homey B, Soto H, Ge N, Catron D (2001). Involvement of chemokine receptors in breast cancer metastasis.. Nature.

[38] Ao M, Franco OE, Park D, Raman D, Williams K (2007). Cross-talk between paracrine-acting cytokine and chemokine pathways promotes malignancy in benign human prostatic epithelium.. Cancer Res.

[39] Mantovani A, Allavena P, Sica A, Balkwill F (2008). Cancer-related inflammation.. Nature.

[40] Seoane J (2008). The TGFBeta pathway as a therapeutic target in cancer.. ClinTranslOncol.

[41] Tang B, Vu M, Booker T, Santner SJ, Miller FR (2003). TGF-beta switches from tumor suppressor to prometastatic factor in a model of breast cancer progression.. JClinInvest.

[42] Flanders KC, Sullivan CD, Fujii M, Sowers A, Anzano MA (2002). Mice lacking Smad3 are protected against cutaneous injury induced by ionizing radiation.. AmJPathol.

[43] Tian F, DaCosta BS, Parks WT, Yoo S, Felici A (2003). Reduction in smad2/3 signaling enhances tumorigenesis but suppresses metastasis of breast cancer cell lines.. Cancer Res.

[44] Stuelten CH, Kamaraju AK, Wakefield LM, Roberts AB (2007). Lentiviral reporter constructs for fluorescence tracking of the temporospatial pattern of Smad3 signaling.. Biotechniques.

[45] Tian F, Byfield SD, Parks WT, Stuelten CH, Nemani D (2004). Smad-binding defective mutant of transforming growth factor beta type I receptor enhances tumorigenesis but suppresses metastasis of breast cancer cell lines.. Cancer Res.

[46] van Waarde MA, van Assen AJ, Kampinga HH, Konings AW, Vujaskovic Z (1997). Quantification of transforming growth factor-beta in biological material using cells transfected with a plasminogen activator inhibitor-1 promoter-luciferase construct.. Anal Biochem.

[47] Stuelten CH, Barbul A, Busch JI, Sutton E, Katz R (2008). Acute wounds accelerate tumorigenesis by a T cell-dependent mechanism.. Cancer Res.

[48] Maroulakou IG, Anver M, Garrett L, Green JE (1994). Prostate and mammary adenocarcinoma in transgenic mice carrying a rat C3(1) simian virus 40 large tumor antigen fusion gene.. Proc Natl Acad Sci U S A.

