# Supplementary figures and images for: Transient Tumor-Fibroblast Interactions Increase Tumor Cell Malignancy by a TGF-β Mediated Mechanism in a Mouse Xenograft Model of Breast Cancer
Source: PLoS One. 2010 Mar 23;5(3):e9832. doi: 10.1371/journal.pone.0009832 (PMC2843748; doi:10.1371/journal.pone.0009832)

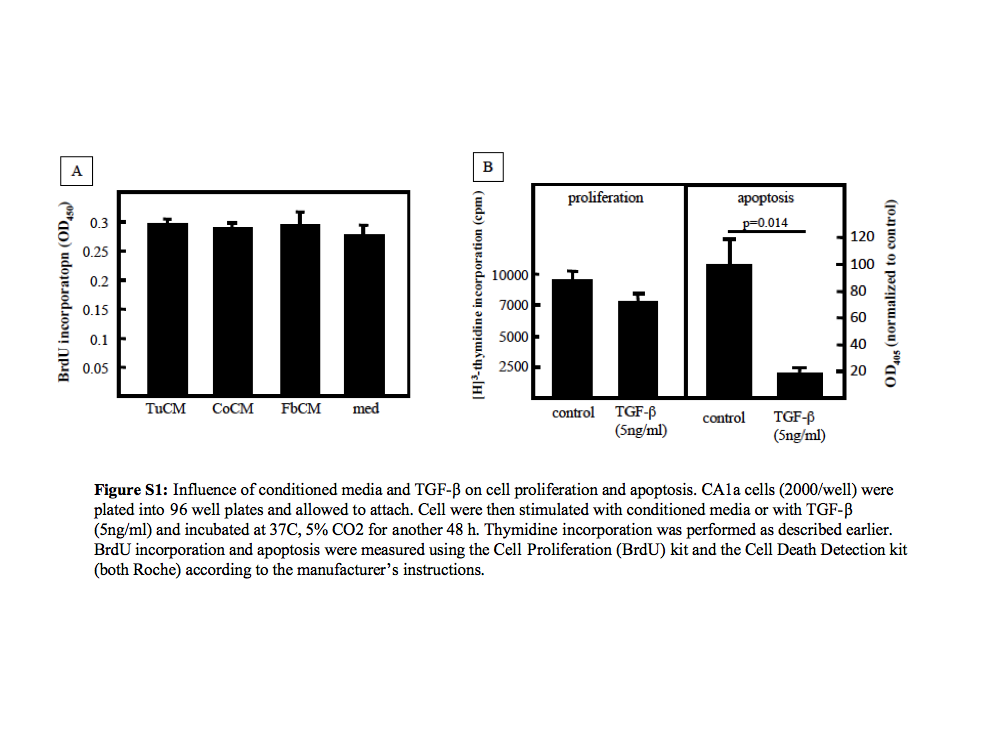

Supplement: Figure S1 — Influence of conditioned media and TGF-beta on cell proliferation and apoptosis. CA1a cells (2000/well) were plated into 96 well plates and allowed to attach. Cell were then stimulated with conditioned media or with TGF-beta (5 ng/ml) and incubated at 37C, 5% CO2 for another 48 h. Thymidine incorporation was performed as described earlier. BrdU incorporation and apoptosis were measured using the Cell Proliferation (BrdU) kit and the Cell Death Detection kit (both Roche) according to the manufacturer's instructions. (0.17 MB TIF) [file pone.0009832.s001.tif]

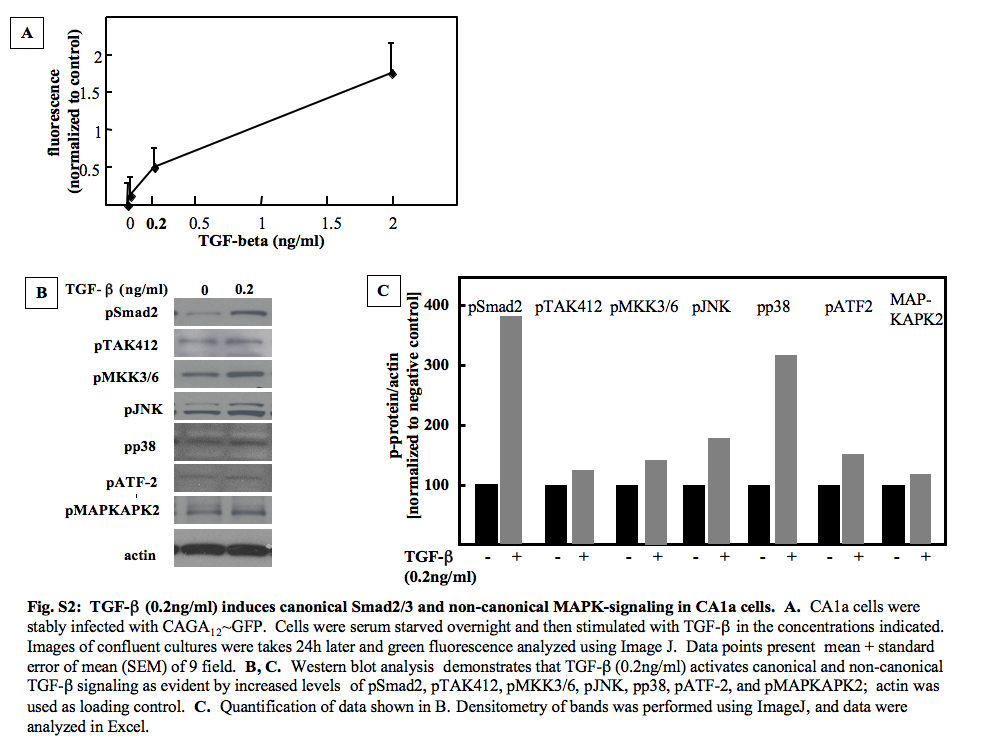

Supplement: Figure S2 — TGF-β (0.2 ng/ml) induces canonical Smad2/3 and non-canonical MAPK-signaling in CA1a cells. A. CA1a cells were stably infected with CAGA12∼GFP. Cells were serum starved overnight and then stimulated with TGF-β in the concentrations indicated. Images of confluent cultures were takes 24 h later and green fluorescence analyzed using Image J. Data points present mean + standard error of mean (SEM) of 9 field. B, C. Western blot analysis demonstrates that TGF-β (0.2 ng/ml) activates canonical and non-canonical TGF-β signaling as evident by increased levels of pSmad2, pTAK412, pMKK3/6, pJNK, pp38, pATF-2, and pMAPKAPK2; actin was used as loading control. C. Quantification of data shown in B. Densitometry of bands was performed using ImageJ, and data were analyzed in Excel. (0.26 MB TIF) [file pone.0009832.s002.tif]

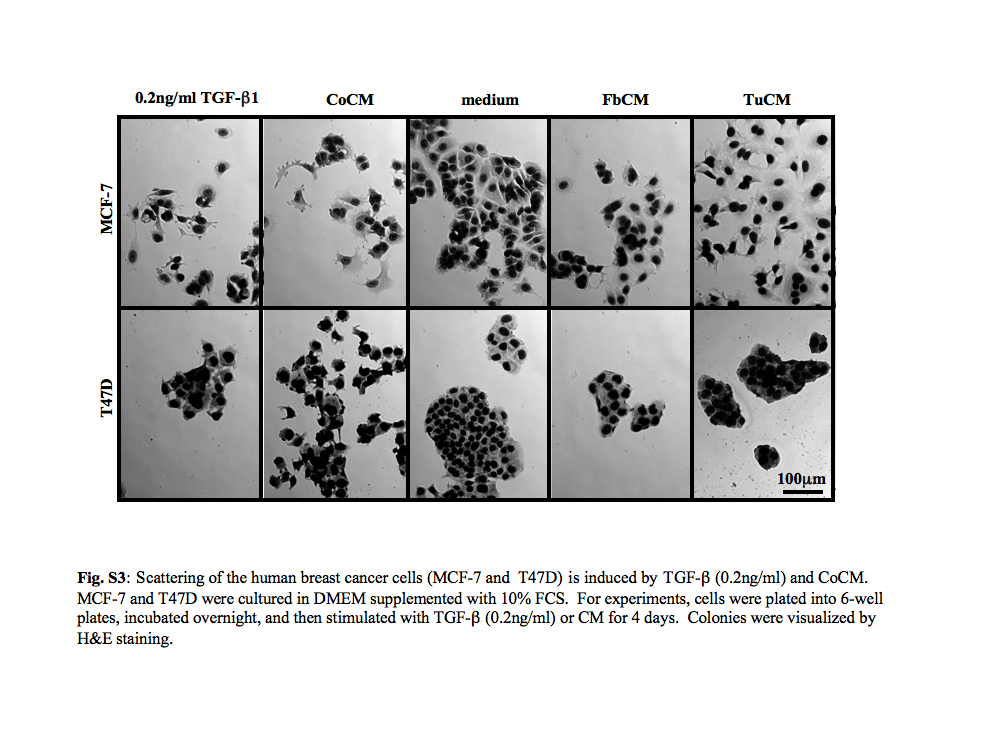

Supplement: Figure S3 — Scattering of the human breast cancer cells (MCF-7 and T47D) is induced by TGF-β (0.2 ng/ml) and CoCM. MCF-7 and T47D were cultured in DMEM supplemented with 10% FCS. For experiments, cells were plated into 6-well plates, incubated overnight, and then stimulated with TGF-β (0.2 ng/ml) or CM for 4 days. Colonies were visualized by H&E staining. well plates, incubated overnight, and then stimulated with TGF-β (0.2 ng/ml) or CM for 4 days. Colonies were visualized by H&E staining (0.51 MB TIF) [file pone.0009832.s003.tif]

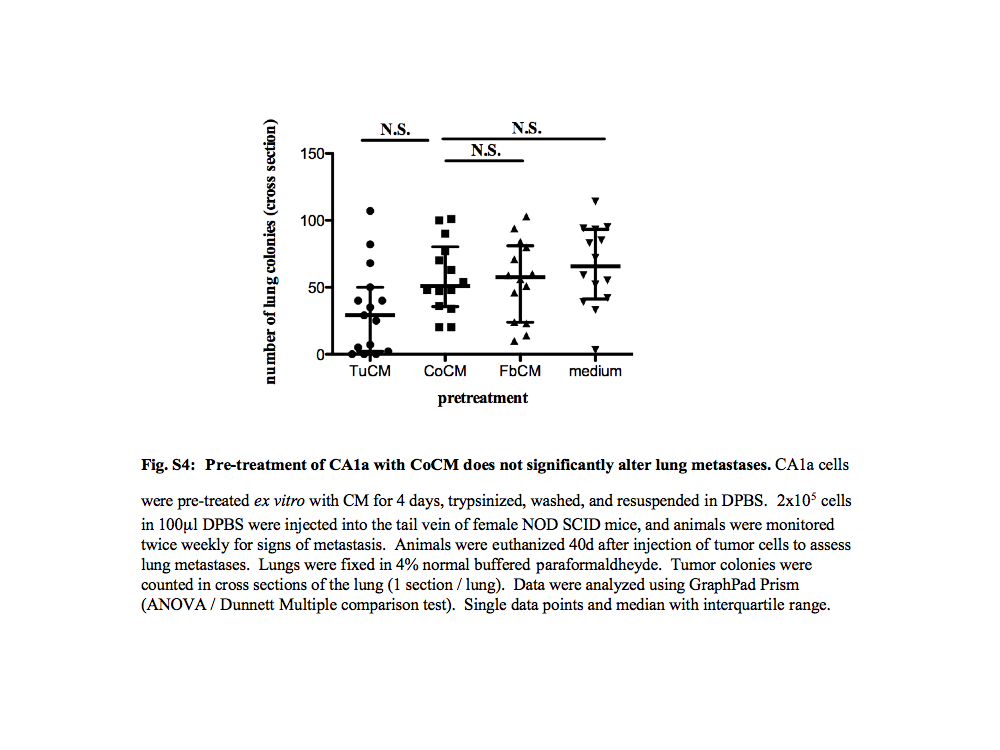

Supplement: Figure S4 — Pre-treatment of CA1a with CoCM does not significantly alter lung metastases. CA1a cells were pre-treated ex vitro with CM for 4 days, trypsinized, washed, and resuspended in DPBS. 2×10^5^ cells in 100 ml DPBS were injected into the tail vein of female NOD SCID mice, and animals were monitored twice weekly for signs of metastasis. Animals were euthanized 40 d after injection of tumor cells to assess lung metastases. Lungs were fixed in 4% normal buffered paraformaldheyde. Tumor colonies were counted in cross sections of the lung (1 section/lung). Data were analyzed using GraphPad Prism (ANOVA/Dunnett Multiple comparison test). Single data points and median with interquartile range. (0.17 MB TIF) [file pone.0009832.s004.tif]
